# Composting of Organic Solid Waste of Municipal Origin: The Role of Research in Enhancing Its Sustainability

**DOI:** 10.3390/ijerph20010312

**Published:** 2022-12-25

**Authors:** Grazia Policastro, Alessandra Cesaro

**Affiliations:** 1 Department of Civil, Architectural and Environmental Engineering, University of Naples Federico II, 80125 Naples, Italy; 2 Telematic University Pegaso, 80132 Naples, Italy

**Keywords:** circular economy, microplastics, persistent contaminants, recovery, resource

## Abstract

The organic solid waste of municipal origin stands as one of the residual streams of greatest concern: the great amounts continuously produced over time as well as its biochemical and physical characteristics require its proper handling via biological processes, pursuing the recovery of material and/or the generation of energy. At the European level, most of the industrial plants treating the organic fraction of municipal solid waste (OFMSW) rely on composting, which is a well-established and reliable process that is easy to operate in different socio-economic contexts. Nevertheless, when regarded in a life cycle perspective as well as in the view of the principles of circular economy underlying waste management, several issues (e.g., the presence of toxic substances in compost) can be recognized as technical challenges, requiring further studies to identify possible sustainable solutions. This work aims at discussing these challenges and figuring out the state of the art of composting in a circular perspective. Firstly, the main mentioned issues affecting compost quality and process sustainability are briefly reviewed. Next, to promote the effective use of composting in light of the circular economy principles, research experiences are critically presented to highlight the current technical challenges concerning the environmental and health impact reduction and possible scientific perspectives to overcome issues affecting the compost quality. Based on the critical analysis of reviewed studies, it emerged that further research should be aimed at unveiling the hazard potential of emerging contaminants as well as to address the understanding of the mechanisms underlying their potential removal during composting. Moreover, the adoption of a multidisciplinary perspective in the design of research studies may play a key role towards the definition of cost-effective and environmentally friendly strategies to overcome the technical issues affecting the process.

## 1. Introduction

The organic fraction of municipal solid waste (OFMSW) is an important share of European household waste [1]. The most recent report of the European Environmental Agency highlights that approximately 85 million tons of biodegradable waste were generated in European Union member states; the greatest portion (around 60%) was composed by food scraps and leftovers, while green waste accounted for approximately 34% [1]. The high amounts as well as the biochemical characteristics of OFMSW make its proper management an issue of great relevance at the European and international levels. The OFMSW is a carbon-rich source that, when improperly managed, may produce severe environmental burdens [2], whose reduction has been pursued by promoting the recovery of resources and energy. In this regard, the comparison of the production of compost from source selected OFMSW and the landfilling of biologically stabilized mechanically sorted OFMSW through a Life Cycle Analysis (LCA) demonstrated that the latter was responsible for the greater impact in terms of global warming [3].

Currently, the principles of the circular economy are underpinning waste management, and an approach directed towards the recovery of materials and/or energy from OFMSW is even more important [1]. To this end, the biological processing of OFMSW appears as the most suitable solution. Recent research advances are addressing the generation of value added biochemicals [4] and energy carriers such as hydrogen [5] and hythane [6]. However, most of them are still in their infancy in terms of being proposed at industrial scale, where composting and anaerobic digestion remain the prevailing options [7]. A comparative review of these processes pointed out that anaerobic digestion may end up being more profitable than composting depending on the process scale, but the former process is definitely more environmentally friendly in terms of greenhouse gas (GHG) emissions, as the biogas may be addressed to energy recovery [7]. Nonetheless, the European Environment Agency recently highlighted that additional environmental benefits can be gained by treating OFMSW with a sequential anaerobic digestion and composting process. Although anaerobic digestion is thus expected to increase significantly, in Europe most of the OFMSW treatment capacity is still provided by composting [1].

**Figure 1:**
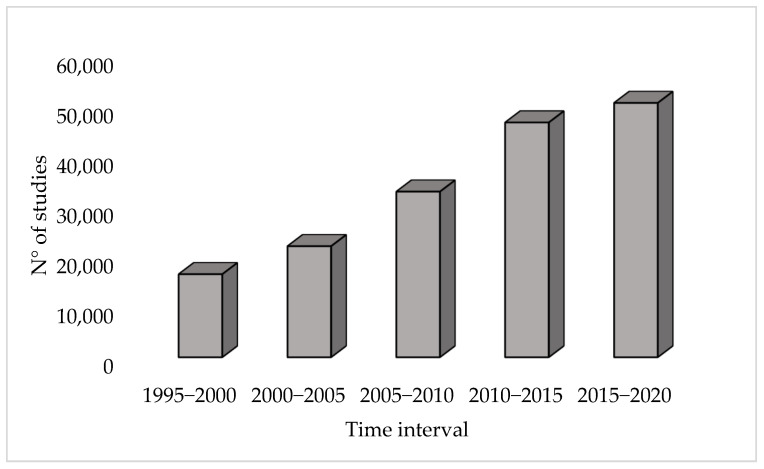
Trend of indexed papers containing the word “composting” from 1995 to 2020 (source of data: Scopus database, accessed in December 2022).

Composting is a well-known biological process, carried out under aerobic conditions, to stabilize organic substrate into a product with fertilizer properties, namely the compost [8]. However, compost is not always a harmless product. Indeed, it may contain various chemical and biological contaminants, and exerting health and/or environmental risks. Such contaminants may expose different population groups to health hazards, including composting plant workers, consumers of compost-treated agricultural products, and children playing on compost-treated parks [8]. The main categories of contaminants which can be contained in the compost are physical pollutants including micro plastics (MPs) and chemical compounds, including persistent organic contaminants such as polycyclic aromatic hydrocarbons (PAHs), polychlorinated biphenyls (PCBs), polychlorinated dibenzo-p-dioxins and -furans (PCDD/Fs), pesticides, and phthalates [9]. Moreover, the possible presence of pathogens leading to microbiological risk has to be considered and properly handled. Furthermore, the composting process can release gaseous compounds of environmental concern such as NH_3_ and GHG (e.g. CO_2_) [9]. Process hazards and environmental impacts depend on composting methods and can be mitigated by the proper operation of the process. The generation of high-quality compost is pivotal to ensuring the effective and safe use of this product on soil due to its agronomic properties. In this regard, research plays a key role in the individuation of challenges and strategies to enhance the process sustainability and perspectives. Indeed, as reported in Figure 1, the number of studies on composting has dramatically increased in recent decades.

The operation of composting in accordance with both the needs of environmental impact reduction and the principle of the circular economy requires a careful analysis, aiming at figuring out the state of the art and identifying the role of research in enhancing its performance. Nonetheless, an updated study meeting these specific needs is absent from the current literature. Indeed, previous works mainly focused on the generic overview of composting challenges and potentials [10,11], as well as on single specific aspects, such as the reduction of nitrogen loss [12], the use of composting products [13], the role of biochar in mitigating GHG emissions in composting [14], composting mathematical models [15], and organic pollutants [16]. In the following paragraphs, industrial composting is briefly outlined, and the more recent research experiences are discussed to individuate main challenges and potential risks as well as to highlight possible strategies and scientific perspectives to promote the sustainability of composting in light of the circular economy principles. After the literature review, an updated and critical analysis of the mentioned challenges, the existing potential strategies as well as possible future research perspectives in this field is provided, in order to deliver a tool for researchers interested in developing new methods and/or improve those currently used, and is aimed at promoting the sustainability of composting.

## 2. Industrial Composting: Main Features and Technical Challenges

### 2.1. Main Features of the Industrial Composting Process

**Figure 2:**
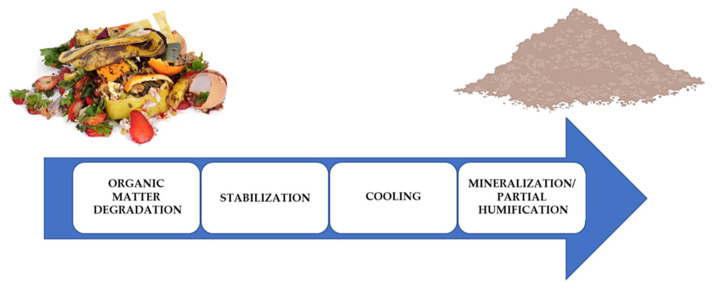
Composting phases.

As shown in Figure 2, the composting process begins as soon as the raw organic materials are mixed together: During the initial stage (organic matter degradation), oxygen and the easily available compounds are consumed by the microorganisms. The temperature of the composting materials then increases rapidly (stabilization phase). As active composting slows, temperature gradually drops (cooling phase) until the compost reaches the environmental temperature. A final curing period usually follows the active composting (mineralization/partial humification) [11].

As reported in Table 1, composting facilities can consist of both closed and open systems.

The former, including biocells, bioreactors and in-vessel composting, require higher capital and operating costs, but allow a better process monitoring. This, in turn, results in lower environmental nuisance as well as in more favorable process conditions, which usually accelerate the biodegradation, shortening the composting period [17,18,19]. Conversely, open systems, such as open windrow and aerated piles, are much less costly: they do not rely on reactors and often air supply is provided by periodically turning the material under processing. In this way, temperature and moisture are also roughly regulated. The lower monitoring extent, in this case, results in longer composting times [20,21] and higher land occupation is also required. In order to overcome the limits of the single composting methods, two-stage processes have also been implemented: in this case, closed systems are used to manage the first biodegradation stages that are more energy intensive and characterized by greater emissions, whereas the final maturation stage is left to open systems [22,23]. Among others, the choice of the composting process depends on the environmental and economic conditions, but it is clear that diverse configurations can be applied in a wide variety of contexts to pursue organic waste recovery [24,25]. Compost generation accounts, indeed, for the interest in composting as a material recovery process pursuing circular economy principles. Moreover, it is recognized as a key process in the food-energy-water nexus, since compost can be used as soil amendment for food production [7].

### 2.2. Technical Challenges

The application of compost on soil brings several benefits, enhancing its main physic-chemical properties [26]. It increases the soil essential levels of both organic matter and nutrients and enhances its bulk density, porosity, water holding capacity, and cation-exchange capacity. Moreover, as a substitute for chemical fertilizer, the use of compost contributes to the reduction of the environmental impacts associated with the production and utilization of chemical fertilizers [27]. However, undesired substances and materials can be found in compost, hindering its safe agronomic use. Compost quality and the emissions of CO_2_ and other GHG are recognized as the main challenges for the sustainability of the process [9]. Both require the identification of proper solutions, and to this end the analysis of the influencing factors is fundamental.

#### 2.2.1. Organic Waste as a Source of Contaminants

The composition and characteristics of the organic substrate destined to composting influences the presence of undesired substances and components in compost [28,29]. The release of CO_2_ is also influenced by the chemical composition of the substrate that drives the biochemical reaction, but little has been reported up to now. Conversely, the issue of organic waste contamination has been significantly debated [28,29].

Non-degradable materials, which may enter composting together with the organic waste, end up in the compost if not removed via mechanical pretreatments or during compost refining stages, with adverse effects on soil [30]. Compost physical contaminants, such as glass, plastics, and synthetic fibers, tend to be incorporated at the soil depth of cultivation, where they may either envelop or act as nucleating agents for mineral grains and organic matter, blocking some pores and potentially reducing water percolation and gas exchange [30].

The presence of physical contaminants can be properly controlled by promoting the source segregation of the organic waste destined for composting. Alvarez et al. [31] studied the correlations between the socio-economic/demographic factors and the percentage of undesirable materials present in biowaste samples to find that in separately collected streams, it ranged between 10 and 20%; conversely, in cities with poor participation in the separate collection schemes, unwanted materials may account up to the 50% of the biowaste. More recently, Echavarri-Bravo et al. [32], through an inter-laboratory trial to evaluate the presence of physical contaminants in compost, posed the issue of their proper detection. The outcomes of their work showed that physical contaminants are heterogeneously present in the source sorted organic waste of municipal origin, and they may require replicate analysis to provide a fair assessment of product quality [32].

Although the improvement of separate collection can enhance the quality of the organic waste, potential miss-sorting and the collection system itself [33] require the mechanical pretreatment of the waste destined to composting [34,35]. On the other hand, despite the beneficial effects of the pretreatment stage on physical contaminants reduction, such pretreatment determines losses of biodegradable materials, mainly through sieving [23], and promotes the reduction of plastic items to micro-plastics (MPs) via shredding and crushing processes. Compost is considered as one of the main sources of MPs in agricultural environments [36]; they may adversely affect the carbon cycle in soil and bring toxic elements (i.e. heavy metals) that have been reported to be associated to MPs during composting [37]. Gui et al. [38] showed the gradual increase of MPs abundance during composting, as well as the change in their shape and size distribution. Therefore, the long-term application of compost onto the soil could result in the accumulation of MPs and consequent impacts on the soil ecosystem.

Although MPs represent a pressing issue, there remains a scarcity of data about their presence, especially for the smaller fractions, due to the difficulties associated with their separation and analysis [39]. However, some attempts have already been made, and a recent study showed that the abundance of MPs tend to increase during the pretreatment of the organic waste destined to composting, whereas manual sorting had no effect on micro-plastics as it aimed at the removal of the larger plastic items, the mechanical shearing and tearing forces exerted by the crushing and pressing steps was the cause of the increase of micro-plastic abundance in the samples entering the composting process [38]. This outcome confirms the key role of source selection in preventing the generation of MPs that can end up in compost. In this regard, studying the refined compost produced from five OFMSW facilities differing for the collection systems and treatment technologies, Edo et al. [40] found that smaller plants with OFMSW door-to-door collection systems produced compost with less plastic of all sizes, whereas compost from big facilities fed by OFMSW from street bin collection displayed the highest contents of plastics. Additionally, the authors reported that no compostable plastic debris was found in the analyzed samples, suggesting that biodegradable polymers that may be present in the incoming waste do not contribute to the spreading of anthropogenic pollution [40]. Biodegradable polymers have been introduced in recent decades to overcome the issue of traditional plastic pollution. The increasing use of such new materials determines their increasing frequent presence in the waste destined to composting. Although, as mentioned, most biodegradable polymers have been found to be suitable for composting, few of them, such as polylactic acid (PLA), may negatively affect the process. More specifically, it has been found that PLA degradation generates lactic acid, which significantly reduces the pH of compost, affecting seed germination [41].

In contrast with non-degradable, physical pollutants, the pre-processing of the organic waste cannot act on the presence of persistent organic contaminants. Polycyclic aromatic hydrocarbons (PAHs), polychlorinated biphenyls (PCBs), polychlorinated dibenzo-p-dioxins and -furans (PCDD/Fs), pesticides, and phthalates have reportedly been found in compost [42,43,44,45], and their fate during the composting process varies depending on the molecular weight, as discussed later in this work.

#### 2.2.2. The Influence of Process Conditions in Driving Composting Sustainability

The composting process aims at organic matter biostabilization, which is mainly affected by the supply of oxygen, the availability of nutrients, the temperature, and the time [46]; the optimization of these operating parameters and conditions are crucial to ensure the biodegradation of organic matter to an adequate extent, determining compost biological stability and maturity.

Compost stability refers to the degree of decomposition of the organic matter, whereas the maturity describes the suitability as soil amendment, indicating the degree of humification [47]. Relevant amounts of organic acids, free ammonia-nitrogen (NH_3_) or other water-soluble compounds that can restrict root development and limit seed germination may be found in unstable composts, and thus the maturity further implies the absence of both phytotoxic compounds in addition to pathogens [47,48,49].

Stability and maturity are a consequence of the proper biodegradation of organic compounds in the presence of nutrients such as nitrogen (N), phosphorous (P), and potassium (K). Carbon and nitrogen are fundamental for microorganisms to gain energy and build new cells, and thus the C/N ratio is used as a process control parameter [50]. During composting, the C/N ratio decreases as a consequence of the decrease of both elements, which occurs at a rate that is higher for C than for N [50]. Several studies reported that a C/N ratio between 25–30 is optimal for proper composting, but values as high as 40 or 50 have been recommended as well [51,52,53]. The C/N ratio may be adjusted by selecting suitable bulking agents, which also play a role in affecting aeration by influencing the porosity of the substrate under composting.

Oxygen supply is the most important parameter in ensuring the proper process development, affecting the microbial activity during the process [54]. Aeration frequency was found to influence the succession of the bacterial community during the industrial food waste composting by affecting both oxygen concentration and the release of various enzymes by these bacteria [54]. Similar outcomes were obtained by Wang et al. [55], who further observed how the aeration rate influenced the leachate production and contributed to the decomposition of toxic substances in the leachate itself.

Cerda et al. [48] reported proper composting development for aeration ranging between 0.2 and 0.6 L/kg_OM_ min, whereas Xu et al. [56], studying bacterial dynamics together with gaseous emissions and humification during the composting of food waste, found that aeration intensities higher than 0.36 L/kg_OM_ min reduced the emissions of GHG and hydrogen sulphide and promoted the production of the humus precursor. The same authors recommended reducing the aeration intensity in the final stages of composting in order to avoid the bacterial consumption of the humus precursors. 

Aeration is not only crucial to providing oxygen supply, as it also helps with regulating the temperature and the moisture in the mass under composting as well as in removing CO_2_ [57]. The moisture, in turn, has been recently pointed out to affect GHG emissions during food and garden waste composting; increasing the moisture content of the waste under composting resulted in more pores filled with water, which determined, in turn, the creation of anaerobic zones where methane (CH_4_) was produced; nevertheless, total nitrous oxide (N_2_O) was found to increase for decreasing moisture content [58]. This condition stands as a technical challenge to be addressed to ensure the sustainability of the process while providing proper aeration conditions. Several studies indeed report that aeration demand for temperature and moisture regulation is much higher than that of biochemical reactions [59,60], and thus excess oxygen is usually supplied in industrial scale plants. 

Most industrialized countries have regulated composting as an OFMSW recovery process, defining specific guidelines. Beyond setting threshold limit values for some target compost parameters, these provide indications about the process operating conditions, including the definition of the waste substrates to be excluded as well as the minimum temperature to be reached to ensure the proper sanitation of the final product [61]. Composting is indeed a self-heating process and temperatures tend to increase in the initial stages as a result of the higher biochemical reaction intensity, and to decline in the final maturation steps, reaching values comparable to the environmental ones. Due to the importance of temperature during composting, this process is divided into the so-called mesophilic, thermophilic and maturation stages, where the former two (i.e. mesophilic and thermophilic) basically refer to the accelerated bio-oxidation phase. In order to produce a hygienic compost, the thermophilic stage should last one week and reach temperatures as high as 55 °C to ensure pathogen destruction [61]. The temperature of the waste under composting is influenced by the external one, so that heating methods have been developed to promote the microbial activity and increase the temperature in cold climate regions [62]. The role of high temperature is indeed fundamental not only for hygiene reasons; it promotes organic matter degradation, shortening the maturity period [63]. Additionally, it may act in controlling the presence of some contaminants. In this regard, Chen et al. [64] reported an almost 43% removal of polystyrene-MPs from sewage sludge after 45 days of hyperthermophilic composting. They concluded that this outcome was due to the excellent bio-oxidation performance exhibited by hyperthermophilic bacteria [64]. However, under thermophilic conditions, the high temperature, humidity and oxygen content could improve the degradation of MPs and increase the release of toxic elements (plasticizer, chlorine and heavy metals). In addition, such conditions could produce the reactive oxygen species that reduces the richness and biodiversity of microbial communities during the conventional composting of cow manure and sawdust [65].

The operating conditions may thus play a role in promoting the contaminant removal; besides MPs, the concentration of selected organic contaminants can be also reduced. This is the case for low molecular weight PAHs, which were observed to decrease up to 90% during composting [66]. Conversely, the concentration of high molecular weight PAHs, PCBs and pesticides was found to remain stable or to increase, likely due to the moisture content reduction during the final steps of composting [66,67,68,69]. Similarly, Graça et al. [69] showed that the use of wood shavings as a bulking agent promoted the proper succession of bacteria during composting, leading to the degradation of low molecular weight PAHs and phthalates, whereas Lin et al. [70] demonstrated the possibility to treat the high concentration of benzophenone during the co-composting of food waste, sawdust and mature compost, reaching a 97% removal efficiency after 35 days of incubation.

It is worth highlighting that composting has been proposed as a bioremediation practice for sites contaminated with organic pollutants, including polycyclic aromatic hydrocarbons, pesticides, and petroleum products [71], as well as polychlorinated dibenzo-p-dioxins (PCDDs) and polychlorinated di-benzofurans (PCDFs) [72]. This indicates a potential for the process to reduce the presence of some toxic compounds (which may be contained in the incoming waste) in the final compost.

## 3. Enhancing Compost Quality and Reducing Gaseous Emissions: The Role of Research

In most industrialized countries where OFMSW recycling practices are well established, the characteristics of compost for its use on soil are clearly identified. Although a lack of uniformity can be observed, the agronomic value (C/N ratio, minimum carbon content) and the presence of heavy metals, inerts and pathogens are usually well established [73]. Similarly, the stability and maturity are already monitored in waste-based composts. The assessment of both biological stability and maturity during the industrial scale composting of the organic fraction of municipal solid waste showed the key role of these parameters in the process monitoring [74]. The analysis of the Dynamic Respiration Index (DRI) over time was found to provide useful indications about the development of the biological stabilization process, although it may not address the correct identification of the possible causes for unstable composts. Similarly, the sole result of phytotoxicity tests cannot provide comprehensive information given the tight link between stability and maturity [74], especially for municipal waste based-compost [75]. The proper monitoring of the selected conventional parameters of stability and maturity may provide further indication about the biodiversity in composting processes [76].

### 3.1. Persistent and/or Emerging Organic Contaminants

The greatest concern about the safe use of compost on soil must be ascribed to those contaminants not yet regulated, and this issue has been largely debated in the scientific literature. The presence of persistent organic contaminants has been investigated to provide a wider perspective, and it was found that they do not usually pose a severe risk. The content of compounds causing dioxin-like effects such as PAHs, PCBs and other chlorinated compounds, analyzed in compost samples collected in 16 European countries, was found to be mostly below the most restrictive limit values [77]. Similarly, Langdon et al. [78] found that many contaminants in composted municipal organic waste samples produced in New South West Australia can be considered as not posing a risk. Nevertheless, for some others, no criteria were available to assess their hazard potential. To this end, a priority ranking was proposed based on the assessment of a risk quotient (RQ) for either ecological or human receptors. This was calculated considering the maximum concentration in soil resulting in two different scenarios of compost application on soil (Table 2).

The need to further explore the adverse effects of most emerging compounds comes along with the need to verify whether the proper adjustment of the composting operating conditions may contribute to their degradation. In this regard, further research should focus on understanding the mechanisms underlying the biologically-mediated oxidation of these organic pollutants during waste composting and, based on scientific literature, similar considerations are raised for microplastics. As mentioned, the establishment of specific temperature profiles may be beneficial for the process [64]. However, further research efforts should be directed towards the understanding of its role on different plastic polymers and size range as well as toward the definition of strategies to reach high temperatures in cold climate regions. In this regard, a suitable solution would be the use of new microbial strains working at psychrophilic conditions, as suggested by Jiang et al. [79]. Generally speaking, the research on microbial communities has indicated that many compounds used for microbial inoculation help to improve the temperature, to extend the high temperature periods, and to enhance kinase activity, chemical composition and enzymes so that more studies are required in this field [11].

Depending on the chemical characteristics and concentration of contaminants, the microbial pools as well as the environmental composting conditions that they contribute to create may differently affect the fate of the contaminant itself [11]. The identification of both optimization strategies and effective monitoring represents other areas of key research to address the technical challenges related to composting.

Process optimization entails the iterative adjustment of the operating conditions to identify those ensuring the sustainable production of a high-quality compost. In this view, an approach based exclusively on an experimental campaign, especially if carried out at industrial scale, may require significant time and effort. Modelling can represent a suitable tool to better understand the composting process [80], identifying the causes of possible failures so as to take prompt action. In this regard, Onwosi et al. [81] briefly reviewed possible statistical and kinetic approaches. The former relies on the use of techniques, including the one-at-a-time approach, factorial design and the fuzzy logic model, which allow the comprehension of the effects of some variables of the process under investigation. On the other hand, the kinetic approach uses mathematical models. The development of effective models is worth studying, with the aim being to address new solutions to be adopted at larger scales.

### 3.2. Gaseous Emissions

The optimization of the composting process is also fundamental to reducing GHG emissions. Recent research advances have focused on the use of semi-permeable membranes coupled with intermittent aeration: under the optimum conditions investigated at industrial scale, a global warming potential (GWP) reduction up to 10% was observed [82] and the carbon dioxide, methane, nitrous oxide, and ammonia emissions outside the membrane during the aeration interval were decreased by 64%, 70%, 55%, and 11%, respectively, compared with that inside the membrane [83].

The optimization of the composting process for GHG may also entail the use of additives [84,85]. Yang et al. [50] proposed the addition of two mineral additives, namely phosphor-gypsum and superphosphate, to reduce gaseous emissions during kitchen waste composting. They found that additives reduced CH_4_ emissions by 80.5–85.8% and decreased NH_3_ emissions by 18.9–23.5%. A decrease in GHG emissions by 7.3–17.4% was also observed. The extensive data analysis carried out by Cao et al. [84] to quantify the impact of different additives on NH_3_ and GHG emissions showed that it was possible to gain greater yields, reducing the loss of total nitrogen as well as the emissions of NH_3_, N_2_O and CH_4_ by 46.4%, 44.5%, 44.6% and 68.5%. The corresponding reduction in the global warming potential was 54.2%. The same authors pointed out that all the additive categories (namely physical chemical and biological) significantly reduced TN loss and NH_3_ emission, although, under optimal conditions, the chemical additives resulted in higher effectiveness [84]. 

Novel additives may thus be identified, or proper activation procedures may be developed to enhance the efficiency of traditional additives with regard to odour and gaseous emissions. It is worth highlighting that, in a circular perspective, carbon dioxide, although contributing to GHG emissions, may be more interestingly captured and recycled. Thomson et al. [86] proposed this option by approaching the composting process optimization in the view of resource recapture with the aim of using CO_2_ and other composting outputs (like heat and the compost itself) within controlled environmental agriculture practices.

In view of enhancing composting sustainability in a circular perspective, its integration within anaerobic digestion facilities has been proved to be a solution. For instance, Di Maria et al. [87] compared composting with integrated anaerobic/aerobic treatments aiming at different energetic use of the biogas produced during the anaerobic stage. Their findings demonstrated that the latter was the preferred option in terms of avoided impacts, especially when considering the upgrading of biogas into biomethane instead of conventional exploitation in co-generators [87]. These results were more recently confirmed by Le Pera et al., [88] highlighting another area of further research based on LCA studies to identify the potential impact of composting and optimize the process by addressing the reduction of emissions.

## 4. Critical Analysis of Reviewed Studies and Concluding Remarks

The reliability of composting and its easy implementation are the main drivers for this process to be the preferred technical solution to manage the organic fraction of municipal solid waste at the European level. Nevertheless, with the shift of the paradigm towards the circular economy, some aspects have emerged as technical constraints limiting the sustainability of this process. The need to ensure the effective and safe use of compost on soil has addressed the discussion on compost quality and the presence of contaminants. Similarly, odour, ammonia and GHG emissions require proper handling to reduce the environmental burdens of the process.

The literature review highlights the central position of these aspects in the scientific debate. Different kinds of persistent organic contaminants have been detected and regarded in the view of the potential risk posed by their release into the environment; similarly, the fate of microplastics during composting has been investigated to verify which process stages contribute the most to their accumulation into the process product. Further studies are needed to unveil the hazard potential of emerging contaminants as well as to address the understanding of the mechanisms underlying their potential removal during composting and to propose novel solutions to be applied at larger scale.

Another issue of concern was found to be related to gaseous emissions: beyond odour control solutions, it is fundamental to reduce the environmental burdens associated with GHG emissions. Novel approaches rely on intermittent aeration and the use of semi-permeable membranes or that of additives, but additional efforts should be devoted to the identification of both the optimal operating conditions and the operating costs to implement these solutions within industrial plants. Moreover, in the view of a circular perspective, any solution addressing the capture and recycling of gaseous compounds may play a pivotal role in the near future. This is particularly true for GHGs such as CO_2_.

It is worth highlighting that research exclusively based on experimental campaigns may turn out to be expensive and time-consuming, so that additional approaches based on either mathematical modelling or life cycle assessment studies may be used to support the identification of the most suitable solutions. However, even though different approaches may serve diverse purposes, adopting a holistic and multidisciplinary perspective in the design of research studies dealing with the composting process may play a key role towards the definition of reliable, cost-effective and environmentally friendly strategies to enhance composting performances. Conversely, despite the essential role of research in this field, the limitation of lab-scale experimental tests has to be overcome via the validation of the most promising findings at real scale.

## Figures and Tables

**Table 1:** Composting systems: main advantages and drawbacks.

System		Advantages	Drawbacks	Ref.
Closed	Biocells; Bioreactors; In-vessel.	Possibility to rely on compact and modular systems; Technologically advanced to properly control the process and the emissions.	High costs; Skilled operator required.	[17,18,19]
Open	Windrow (i.e., Turning piles); Aerated static piles.	Low capital costs; Easy operation; Basically adaptable to different territorial contexts.	High land requirement; Poor emission control; Long retention times.	[20,21]

**Table 2:** Prioritization of selected contaminants based on the risk assessment conducted by Langdon et al. [78]).

Priority Group	Risk Receptor	Contaminant of Potential Concern	RQ_max_ (10 t/ha)	RQ_max_ (140 t/ha)
Very high	Ecological	Phenol	3.5	45
	Ecological	Dibutyl phthalate	1.8	23
	Ecological	Commercial penta-BDE	2.3	30
	Human health	Total PBDEs	5.5	70
High	Ecological	DEHA	0.45	5.7
	Ecological	BPA	0.14	1.7
Medium	Ecological	DEHP	0.11	23
	Ecological	DBT	0.11	1.5
Low	Ecological	Benzyl butyl phthalate	0.0092	0.12
	Human health	DEHP	0.046	0.58

## Data Availability

The data analyzed during this work are available in the cited literature.
